# Supplementary material for: Measuring the relationship between museum attributes and visitors: An application of topic model on museum online reviews
Source: PLoS One. 2024 Jul 8;19(7):e0304901. doi: 10.1371/journal.pone.0304901 (PMC11230551; doi:10.1371/journal.pone.0304901)
Supplement: S1 File — (ZIP) [file pone.0304901.s001.zip › Support Information/Data analysis process.docx]

1. Descriptive statistics

The situation of the variables was analysed using descriptive statistics and the results are shown in the table below:

Table of descriptive statistics

| Title | Number of cases | Minimum value | Maximum value | Mean value | Standard deviation |
| --- | --- | --- | --- | --- | --- |
| Experiencewithkids | 102728 | 0.0040616330 | 0.6764368180 | 0.0569488145 | 0.0584794306 |
| Friendlystaff | 102728 | 0.0040589700 | 0.5882624730 | 0.0374814983 | 0.0369273668 |
| Londonmuseums | 102728 | 0.0042450130 | 0.7190685930 | 0.1223033890 | 0.0803096894 |
| Entrancehall | 102728 | 0.0024934390 | 0.6791644120 | 0.0287082875 | 0.0358107237 |
| Queuing | 102728 | 0.0024934390 | 0.6791644120 | 0.0287082875 | 0.0358107237 |
| Collections | 102728 | 0.0014688310 | 0.7738736980 | 0.0438260053 | 0.0630627274 |
| Culturalrelics | 102728 | 0.0016810890 | 0.6745812060 | 0.0353442983 | 0.0563815181 |
| Crowds | 102728 | 0.0011954360 | 0.8437883480 | 0.0507192889 | 0.0838763698 |
| Visitingtime | 102728 | 0.0042653480 | 0.6790666140 | 0.0636184533 | 0.0558587181 |
| Museumpreference | 102728 | 0.0067193820 | 0.6787046930 | 0.1285532640 | 0.0870031129 |
| Praises | 102728 | 0.0045582540 | 0.5958782160 | 0.0448894064 | 0.0444247336 |
| Location | 102728 | 0.0009629710 | 0.5769426330 | 0.0525224668 | 0.0485222916 |
| Artist | 102728 | 0.0011143210 | 0.7621261220 | 0.0480481461 | 0.0736512219 |
| Shopping | 102728 | 0.0005910860 | 0.8813342160 | 0.0263820603 | 0.0672047643 |
| GuidedTours | 102728 | 0.0008954030 | 0.5938849190 | 0.0470450274 | 0.0460187915 |
| Bookingandtickets | 102728 | 0.0019730810 | 0.7194343310 | 0.0353964888 | 0.0550419983 |
| Unfriendlystaff | 102728 | 0.0043344070 | 0.6536997950 | 0.0526724156 | 0.0559772168 |
| Caféanddining | 102728 | 0.0030424700 | 0.8985018500 | 0.0509253007 | 0.0806572132 |
| Enjoyableexperience | 102728 | 0.0041080370 | 0.7483948620 | 0.0538338351 | 0.0614667798 |
| Satisfaction | 102728 | 1 | 5 | 4.62 | 0.706 |

1. Data were first analysed for reliability and validity
2. Reliability analysis

The data were analysed for reliability and validity using Cronbach's Alpha for data reliability. From the table below, it can be seen that the dimensions of the data and the total Cronbach Alpha coefficient are greater than 0.7, indicating that the overall reliability of the data is high (Cronbach Alpha coefficient generally reaches more than 0.7, which can be a response to the high reliability of the data, which can be further analysed in-depth correlation with the data).

Reliability analysis table

| Dimension | Cronbach Alpha | No. |
| --- | --- | --- |
| Core offering | 0.903 | 3 |
| Peripheral services | 0.957 | 9 |
| Ambiance | 0.897 | 4 |
| Personal Experience | 0.939 | 3 |
| Total | 0.951 | 19 |

1. Validity Analysis

Next, validity analysis was performed using factor analysis. In the validity analysis, generally speaking, the data is suitable for factor analysis when the KMO value remains above 0.7. As can be seen from the table below, the value obtained from the KMO test is 0.945>0.7 and the Bartlett's test of sphericity Sig is 0.000<0.001, which is significantly valid at the 0.001 level and suitable for factor analysis

KMO and Bartlett's test

| KMO Number of Sampling Suitability Measure. | | 0.945 |
| --- | --- | --- |
| Bartlett's test | Approximate cardinality | 2077176.146 |
|  | Degrees of freedom | 171 |
|  | Significance | 0.000 |

Through further in-depth analysis, it can be concluded from the table below that the total variance explained by the extracted factors is 83.986%, i.e. the factors have a better explanatory ability and are able to retain the original data information in a more complete manner.

Total Variance Explanation

| component | Initial eigenvalues | | | Extracting the sum of squared loads | | | Rotating load sum of squares | | |
| --- | --- | --- | --- | --- | --- | --- | --- | --- | --- |
|  | Total | Variance per cent | Cumulative % | Total | Variance Percentage | Cumulative %  Total | Total | Variance Percentage | Cumulative % |
| 1 | 10.823 | 56.965 | 56.965 | 10.823 | 56.965 | 56.965 | 6.828 | 35.938 | 35.938 |
| 2 | 2.324 | 12.233 | 69.197 | 2.324 | 12.233 | 69.197 | 3.599 | 18.942 | 54.880 |
| 3 | 1.546 | 8.135 | 77.333 | 1.546 | 8.135 | 77.333 | 2.779 | 14.624 | 69.504 |
| 4 | 1.264 | 6.653 | 83.986 | 1.264 | 6.653 | 83.986 | 2.752 | 14.482 | 83.986 |
| 5 | 0.515 | 2.712 | 86.698 |  |  |  |  |  |  |
| 6 | 0.309 | 1.628 | 88.326 |  |  |  |  |  |  |
| 7 | 0.282 | 1.485 | 89.811 |  |  |  |  |  |  |
| 8 | 0.270 | 1.422 | 91.234 |  |  |  |  |  |  |
| 9 | 0.246 | 1.297 | 92.530 |  |  |  |  |  |  |
| 10 | 0.231 | 1.216 | 93.747 |  |  |  |  |  |  |
| 11 | 0.186 | 0.980 | 94.727 |  |  |  |  |  |  |
| 12 | 0.177 | 0.932 | 95.658 |  |  |  |  |  |  |
| 13 | 0.168 | 0.884 | 96.542 |  |  |  |  |  |  |
| 14 | 0.161 | 0.847 | 97.390 |  |  |  |  |  |  |
| 15 | 0.115 | 0.607 | 97.997 |  |  |  |  |  |  |
| 16 | 0.113 | 0.597 | 98.594 |  |  |  |  |  |  |
| 17 | 0.103 | 0.541 | 99.135 |  |  |  |  |  |  |
| 18 | 0.094 | 0.497 | 99.632 |  |  |  |  |  |  |
| 19 | 0.070 | 0.368 | 100.000 |  |  |  |  |  |  |

Based on the factor loadings in the table below it can be seen that the questions fall into the corresponding dimensions. This indicates that the validity of the data is good and the data obtained can be used for further analysis. Overall looking at the whole data, the reliability and validity is high, reliable and valid and can be used for research and analysis.

Rotated component matrix

|  | component | | | |
| --- | --- | --- | --- | --- |
|  | 1 | 2 | 3 | 4 |
| Guided Tours | 0.882 |  |  |  |
| Location | 0.859 |  |  |  |
| Café and dining | 0.856 |  |  |  |
| Friendly staff | 0.850 |  |  |  |
| Shopping | 0.849 |  |  |  |
| Booking and tickets | 0.824 |  |  |  |
| London museums | 0.820 |  |  |  |
| Museum preference | 0.776 |  |  |  |
| Unfriendly staff | 0.722 |  |  |  |
| Queuing |  | 0.889 |  |  |
| Crowds |  | 0.882 |  |  |
| Entrance hall |  | 0.836 |  |  |
| Visiting time |  | 0.820 |  |  |
| Enjoyable experience |  |  | 0.858 |  |
| Praises |  |  | 0.851 |  |
| Experience with kids |  |  | 0.829 |  |
| Collections |  |  |  | 0.866 |
| Cultural relics |  |  |  | 0.848 |
| Artist |  |  |  | 0.847 |

1. Hypothesis testing

Validated factor analysis

Validated factor analysis tests were conducted. Generally speaking, the standardised factor loadings of the data need to be greater than 0.6, the CR of the combination of dimensions needs to be greater than 0.7, and the average variance extracted (AVE) needs to be greater than 0.5. This indicates that the structural validity of the data is better, and it can be used for structural equation modelling. Firstly, the validated factor analysis model was plotted.


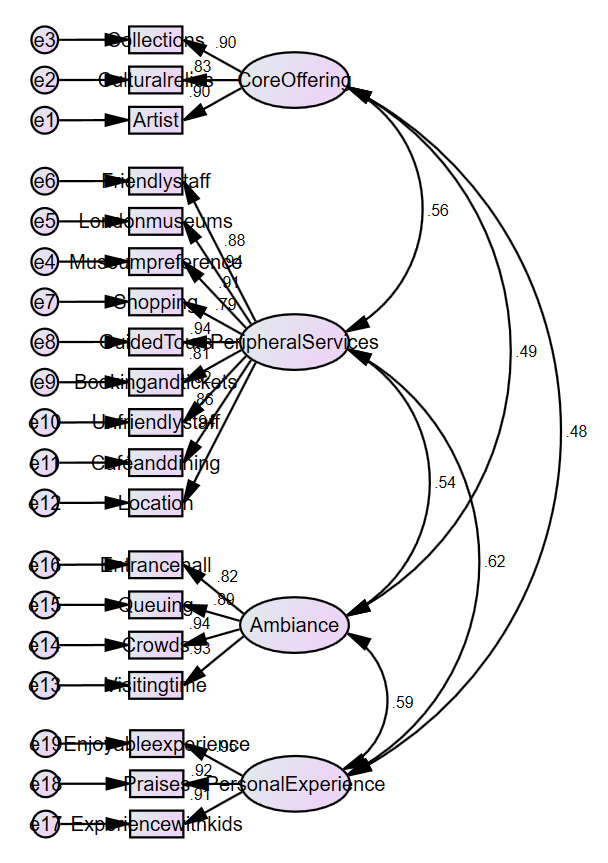


According to the table below it can be seen that the fit of the validated factor analysis models all reached the desired value, indicating that the models were fitted well.

Structural Equation Fit Tables

| Model Fit Indicators | RMR | NFI | IFI | TLI | CFI | RMSEA |
| --- | --- | --- | --- | --- | --- | --- |
| Desired value | ≤0.080 | ≥0.900 | ≥0.900 | ≥0.900 | ≥0.900 | ≤0.10 |
| Fit value | 0.001 | 0.932 | 0.932 | 0.920 | 0.932 | 0.097 |

According to the table below it can be seen that the standardised factor loadings for each question item and the CR and AVE values for each dimension in the validated factor analysis met the criteria, indicating that the data had good structural validity.

Validation factor analysis table

| Dimension | Item | Estimate | CR | AVE |
| --- | --- | --- | --- | --- |
| Core offering | Artist | 0.901 | 0.909 | 0.770 |
|  | Culturalrelics | 0.826 |  |  |
|  | Collections | 0.903 |  |  |
| Peripheral services | Museumpreference | 0.912 | 0.968 | 0.774 |
|  | Londonmuseums | 0.938 |  |  |
|  | Friendlystaff | 0.883 |  |  |
|  | Shopping | 0.795 |  |  |
|  | GuidedTours | 0.940 |  |  |
|  | Bookingandtickets | 0.813 |  |  |
|  | Unfriendlystaff | 0.823 |  |  |
|  | Caféanddining | 0.862 |  |  |
|  | Location | 0.936 |  |  |
| Ambiance | Visitingtime | 0.930 | 0.943 | 0.805 |
|  | Crowds | 0.937 |  |  |
|  | Queuing | 0.893 |  |  |
|  | Entrancehall | 0.824 |  |  |
| Personal Experience | Experiencewithkids | 0.913 | 0.949 | 0.861 |
|  | Praises | 0.920 |  |  |
|  | Enjoyableexperience | 0.950 |  |  |

Finally, discriminant validity analysis is carried out, in general, the correlation between the dimensions needs to be less than the square root of the AVE of each dimension, which can indicate that the intra-dimensional correlation is higher than the correlation between the dimensions, which indicates that the data has a better discriminant validity. According to the table below, it can be seen that the correlation of each dimension is less than the square root of the AVE of each dimension, which indicates that the data has better discriminant validity and can be used for structural equation analysis.

Discriminant validity scale

|  | Core offering | Peripheral services | Ambiance | Personal Experience |
| --- | --- | --- | --- | --- |
| Core offering | 0.877 |  |  |  |
| Peripheral services | 0.561 | 0.880 |  |  |
| Ambiance | 0.485 | 0.544 | 0.897 |  |
| Personal Experience | 0.483 | 0.623 | 0.589 | 0.928 |

1. AMOS Structural Equation Path Analysis

Path analysis is utilised and used to explore the relationship between the independent and dependent variables. Firstly, the structural equation model was developed based on the theoretical assumptions as follows:


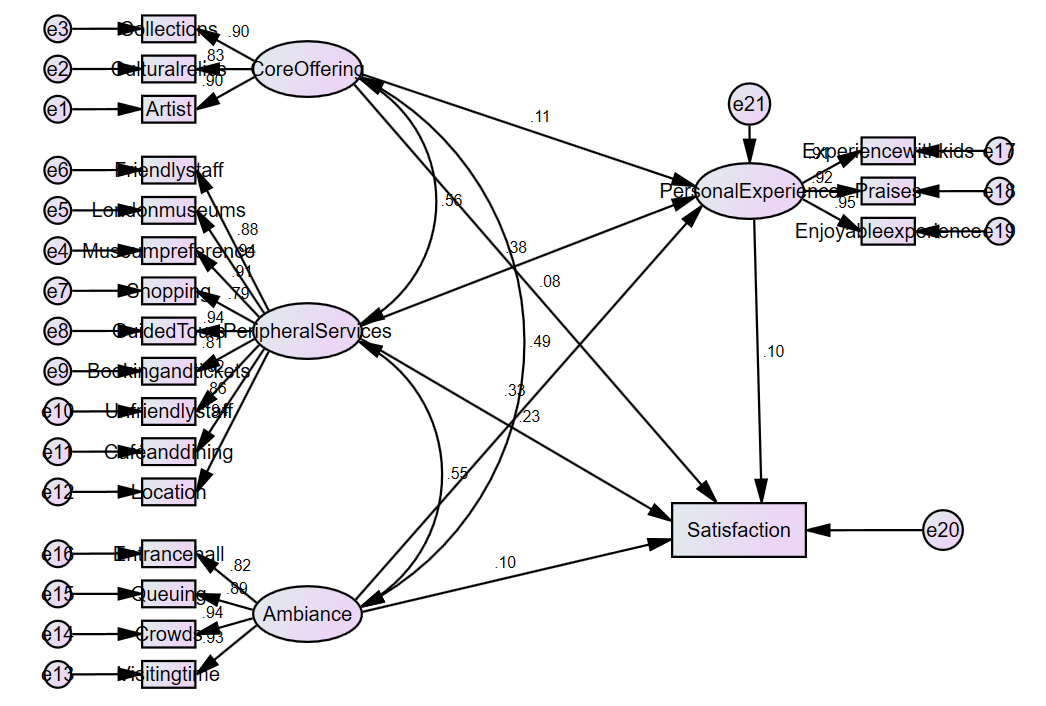


According to the table below it can be seen that the fit of the validated factor analysis models all reached the desired value, indicating that the models were fitted well.

Table of structural equation fits

| Model Fit Indicators | RMR | NFI | IFI | TLI | CFI | RMSEA |
| --- | --- | --- | --- | --- | --- | --- |
| Desired value | ≤0.080 | ≥0.900 | ≥0.900 | ≥0.900 | ≥0.900 | ≤0.10 |
| Value Fit value | 0.001 | 0.932 | 0.932 | 0.920 | 0.932 | 0.097 |

The path analysis test was performed and the following table was obtained.

| **Path** | **Estimate** | **β** | **S.E.** | **C.R.** | **P** | **Supported?** |
| --- | --- | --- | --- | --- | --- | --- |
| Core offerings→Personal experience | 0.087 | 0.109 | 0.003 | 32.582 | *** | Yes |
| Peripheral services→Personal experience | 0.258 | 0.384 | 0.002 | 111.908 | *** | Yes |
| Ambiance→Personal experience | 0.336 | 0.327 | 0.003 | 100.890 | *** | Yes |
| Core offerings→Satisfaction | 0.870 | 0.082 | 0.043 | 20.392 | *** | Yes |
| Peripheral services→Satisfaction | 2.025 | 0.228 | 0.039 | 52.360 | *** | Yes |
| Ambiance→Satisfaction | 1.293 | 0.095 | 0.056 | 23.244 | *** | Yes |
| Personal experience→Satisfaction | 1.371 | 0.104 | 0.058 | 23.796 | *** | Yes |
